# S11-1 The cooperative planning approach in health promotion: Theoretical foundation, theoretical classification and key elements

**DOI:** 10.1093/eurpub/ckac093.055

**Published:** 2022-08-29

**Authors:** Peter Gelius

**Affiliations:** Department of Sport Science and Sport, FAU Erlangen-Nürnberg, Erlangen, Germany

**Keywords:** health promotion theory, participatory approaches, knowledge co-creation, scaling-up, sustainability

## Abstract

**Background:**

Implementing effective physical activity (PA) interventions into routine practice is challenging once research funds run out, and only a minority of programs are successfully moved from research to practice settings and become embedded in a system. Participatory approaches are seen as a means to overcome this trap and sustainably implement and scale-up programs. This presentation provides methodological and methodological overview of the cooperative planning approach (CP), a participatory method increasingly used in PA promotion in the last years.

**Methods:**

We reviewed the literature to trace the origins of the CP approach, outline its basic theoretical foundations, and summarize its central components and procedures. In addition, we compiled a structured overview of previous CP projects to highlight potential application contexts of the approach. Building on the results of a scoping review, we position the CP method within the body of existing participatory approaches based on Arnstein's ladder of participation.

**Results:**

From a theoretical point of view, CP can be traced to the literature on knowledge co-creation and participatory research. It bears conceptual similarities with various organization-based planning methods. There are several distinctive characteristics that set it apart, including (a) the heterogeneity and expertise of participants, (b) a specific process sequence, (c) key success indicators, and (d) structured outputs. Variations of the approach have been successfully employed in sports development and physical activity promotion for target groups across the life-course. Positioning CP within the universe of existing approaches shows that it offers comparatively high levels of participation, is focused on later stages of the implementation process, and is well-suited to be combined with other methods of participation (e.g. citizen science).

**Conclusions:**

The CP approach constitutes an alternative to existing knowledge co-creation and participatory approaches that may help overcome the problem of the pilot project trap. It can be easily adapted to different contexts but is especially suitable for settings where the development of specific measures for PA promotion is required. However, a successful implementation of the CP process depends on a number of prerequisites, e.g. sufficient resources and the engagement of key persons identified as ‘champions'.

